# Non-equilibrium landscape and flux reveal the stability-flexibility-energy tradeoff in working memory

**DOI:** 10.1371/journal.pcbi.1008209

**Published:** 2020-10-02

**Authors:** Han Yan, Jin Wang

**Affiliations:** 1 State Key Laboratory of Electroanalytical Chemistry, Changchun Institute of Applied Chemistry, Chinese Academy of Sciences, Changchun, Jilin, P.R. China; 2 Department of Chemistry and Physics, State University of New York at Stony Brook, Stony Brook, NY, USA; Det Medisinske Fakultet, NTNU, NORWAY

## Abstract

Uncovering the underlying biophysical principles of emergent collective computational abilities, such as working memory, in neural circuits is one of the most essential concerns in modern neuroscience. Working memory system is often desired to be robust against noises. Such systems can be highly flexible for adapting environmental demands. How neural circuits reconfigure themselves according to the cognitive task requirement remains unclear. Previous studies explored the robustness and the flexibility in working memory by tracing individual dynamical trajectories in a limited time scale, where the accuracy of the results depends on the volume of the collected statistical data. Inspired by thermodynamics and statistical mechanics in physical systems, we developed a non-equilibrium landscape and flux framework for studying the neural network dynamics. Applying this approach to a biophysically based working memory model, we investigated how changes in the recurrent excitation mediated by slow NMDA receptors within a selective population and mutual inhibition mediated by GABAergic interneurons between populations affect the robustness against noises. This is realized through quantifying the underlying non-equilibrium potential landscape topography and the kinetics of state switching. We found that an optimal compromise for a working memory circuit between the robustness and the flexibility can be achieved through the emergence of an intermediate state between the working memory states. An optimal combination of both increased self-excitation and inhibition can enhance the flexibility to external signals without significantly reducing the robustness to the random fluctuations. Furthermore, we found that the enhanced performance in working memory is supported by larger energy consumption. Our approach can facilitate the design of new network structure for cognitive functions with the optimal balance between performance and cost. Our work also provides a new paradigm for exploring the underlying mechanisms of many cognitive functions based on non-equilibrium physics.

## Author summary

Working memory is often associated with stimulus-selective persistent activities in the prefrontal cortex. The nature of a memory storage system indicates that the persistent states should be robust to the noise. However, the brain is inherently noisy. Furthermore, sometimes tilting the balance in favor of the increased flexibility rather than the robustness is required according to the environmental conditions and behavioral task demands. This raises the question of what the precise mechanism is for the interplay between the robustness and flexibility in noisy working memory systems. Applying the non-equilibrium potential landscape and flux approach, we found that the network architecture plays a crucial role in determining the robustness against noises and the flexibility to external stimulus. In contrast to the conventional wisdom that there is a conflict between robustness and flexibility, we found that a combination of both increased self-excitation and mutual inhibition can enhance the flexibility to external signals without significantly reducing the robustness to the random fluctuations. The key element of the underlying mechanism for achieving good performance in working memory is the emergence of the new intermediate state. Furthermore, the good performance in working memory is supported by the larger energy expenditure.

## Introduction

The brain is a complex system which can perform different cognitive/“computational” tasks [1–4]. Such computational abilities can emerge as collective properties of networks having an enormous number of neurons. Understanding how the cooperative effects of neuronal interactions can achieve physiological or cognitive functions is one of the most essential questions that computational neuroscience aims to address.

Working memory, which is the ability to transiently hold and manipulate information for short periods of time, is essential for a variety of cognitive behaviors, including learning and decision-making [5–7]. The prefrontal cortex (PFC) is identified as the brain region the most closely linked to working memory [8–13]. During a memory period, the prefrontal cortex neurons show elevated persistent activities triggered by a remembered stimulus [9, 11–16]. In general, positive feedback is thought to be responsible for generating persistent states [11, 14, 17–19]. As a memory storage system, these persistent states are desired to be robust to noise [19]. However, noise is ubiquitous in the brain, e.g. intrinsic random fluctuations and external distraction [20–22]. The intrinsic random fluctuations can be caused by stochastic inputs and spikes receiving from different neurons, small copy numbers of proteins and ionic species in individual neurons and some probability of failures of synapses transmit signals. Even with persistent states, the animal is highly likely to make an error. This then raises the questions of what the general principles are for maintaining robust working memories in noisy biological systems. Furthermore, one sometimes needs to tilt the balance in favor of increased flexibility rather than the robustness according to the environmental conditions and behavioral task demands [10, 23, 24]. It is crucial to understand if robustness and flexibility can be implemented in a single neural circuit. Moreover, it is generally assumed that there is a conflict between the robustness and flexibility in working memory. Does an optimal configuration between them exist? The underlying biophysical mechanism is still unclear.

In a conventional way, previous computational modeling works investigated the robustness and further flexibility in working memories by tracing the neuronal activity trajectories in a limited time scale [13, 25]. The robustness quantified in this way depends on the volume of statistical data. Moreover, the evolution of individual trajectories can only reflect the local properties rather than the global natures of the system. Can we obtain the global natures of collective phenomena in complex systems without replying only on tracing the dynamics of each elementary component? In fact, we can be inspired from thermodynamics and statistical mechanics. The collective properties of a complex system made from a large number of simple elements can be described by the thermodynamics quantities, such as internal energy, free energy and entropy. Hopfield explored the computational properties in neural circuits by constructing an “energy function”, which can provide a clear dynamical picture of how neural circuits implement their cognitive functions, e.g. memory storage and retrieval [1, 2]. From this energy perspective, the global natures of systems can be quantified through the underlying energy landscape. However, the energy function in the original Hopfield model can only be constructed for neural circuits under symmetrical synaptic connections. For more realistic biological neural circuits under asymmetrical connections, the original Hopfield model fails to apply.

Furthermore, as other biological systems, neural circuits are dissipative, consuming energy to perform different vital functions [26, 27]. In such non-equilibrium systems having exchanges of energies with the environment, the dynamics are not determined only by the underlying energy landscapes but also the steady state probability flux quantifying the degree of detailed balance breaking or non-equilibriumness [28–31]. The crucial role of energy cost in cognitive processes such as the modulation of robustness versus flexibility in working memory is unknown either. A question then arises: does the performance in working memory benefit from larger energy supply?

To address these questions, we developed a general non-equilibrium landscape and flux approach [28, 29, 32–34] to study a biophysically based working memory model [13, 35–37]. We uncovered that the network architecture(both self-excitation within one selective population and mutual inhibition between two populations) plays a crucial role in determining the robustness against noises through quantifying the underlying non-equilibrium potential landscapes. The robustness against noises can be enhanced by stronger mutual inhibition. Stronger self-excitation can also induce more robust memories until an intermediate state emerges. The intermediate state that results from increasing the self-excitation reduces the difficulty of jumping from the present memory attractor to the new one through lowing the corresponding barriers. The emergence of such intermediate state suggests a possible mechanism to achieve an optimal compromise for a working memory circuit between robustness and flexibility. We found a combination of both increased self-excitation and inhibition can enhance the flexibility to a new stimulus without significantly reducing the robustness to random fluctuations. Moreover, we found that sufficient energy supply is crucial for the robustness in working memory and necessary for the optimality between robustness and flexibility.

## Methods and materials

### Theoretical framework

Due to the stochastic nature of a realistic neural network, focusing on the probabilistic evolution of the whole system is more appropriate than following the individual trajectories in finite times for characterizing the dynamics globally. In addition, neural networks are open systems, which constantly exchange energy and information with the environment. Therefore, it is natural to explore the neural networks from the perspective of non-equilibrium statistical mechanics. We developed a non-equilibrium potential landscape and flux theory for the general neural networks to address the issues of global stability, function and robustness [28]. We can start with a set of Langevin equations describing the stochastic dynamics of neural networks: $\frac{dx}{dt}=F\left(x\right)+\zeta$. *ζ* represents the stochastic fluctuations, which are assumed to follow a Gaussian distribution with autocorrelations specified by < *ζ*(**x**, *t*) *ζ*(**x**, *t*′) > = 2**D**(**x**)*δ*(*t* − *t*′). Here **D**(**x**) is the diffusion coefficient matrix giving the magnitude of the fluctuations. *δ*(*t*) is a delta function. The evolution of the probability distribution can be quantified by solving the corresponding local probability conservation law: ∂*P*(**x**, *t*)/∂*t* = −∇⋅**J**, which means that the change of the probability is from the net probability flux. Here the probability flux **J** = (**F**(**x**)*P(**x**, *t*)) + ∇⋅(∇⋅(**D** *P*(**x**, *t*))) is determined by both the deterministic force and stochastic fluctuations in addition to the probability. When plugging in the flux expression to the local probability conservation law, we can obtain a Fokker-Planck equation for the probability evolution ∂*P*(**x**, *t*)/∂*t* = −∇⋅(**F**(**x**)*P(**x**, *t*)) + ∇⋅(∇⋅(**D***P*(**x**, *t*))). Furthermore, the steady state probability distribution *P*_*ss*_ can be solved from ∇⋅(**F**(**x**)*P_*ss*_(**x**))−∇⋅(∇⋅(**D***P*_*ss*_(**x**))) = 0. Finally, we can quantify the potential landscape by the relationship *U* = −*lnP*_*ss*_(**x**), which is analogous to equilibrium systems where the potential landscape is related to the equilibrium distribution through the Boltzman law.

Quantifying the topography of the potential landscape through the steady state probability distributions, which reflect the relative weight of each state, can help to account for the stability of the functional states(attractors). In addition, the mean first passage time(MFPT) for representing the average kinetic time of switching from one stable state to another can also describe the stability of stable attractor states. The mean first passage time *τ* from any state to a given final state can be obtained by solving the following equation:**F**⋅∇*τ* + **D**⋅∇⋅∇*τ* = −1 [38]. The boundary condition is taken as an absorbing boundary condition *τ* = 0 at the final state and reflecting boundary conditions *n*⋅∇*τ* = 0 for the outer boundary.

A distinguishing feature of non-equilibrium systems is the presence of nonvanishing steady-state flux. Different from the equilibrium system whose driving force can be expressed to a gradient of an energy function, the neural network as a non-equilibrium system is driven by both the nonvanishing steady-state irreversible probability flux that signifies the violation of detailed balance and the gradient of the potential landscape in the state space:**F** = **J**_*ss*_/*P*_*ss*_−**D**⋅∇*U* [28–33]. Moreover, the non-equilibrium steady states can be maintained by the constantly exchanging matter, energy or information with the environment, where the nonvanishing steady-state flux plays a crucial role.

Biological systems, such as neural circuits, consume energy to perform different vital functions [32, 39]. Recent work shows that the free energy dissipation rate is related to the entropy production rate(epr) in a biochemical system where energy originates from ATP hydrolysis [40, 41]. For an open system, we can define a generalized free energy *H*. The corresponding temporal evolution satisfies *dH*/*dt* = *F*_*in*_ − *F*_*dis*_. Here the *F*_*in*_, which is also termed as the house keeping heat [42], represents the free energy input for maintaining the steady state and the free energy dissipation rate *F*_*dis*_ equals the entropy production rate(epr) multiplied by the temperature(T) [40]. At the non-equilibrium steady state, *F*_*in*_ = *F*_*dis*_. For a continuous dynamical system that can be described by the Fokker-Planck equation, the free energy dissipation rate(entropy production rate) can be calculated as: *epr* = ∫*d* **x**(**J**⋅**D**^−**1**^⋅**J**)/*P* [26, 27, 32, 43, 44]. The flux as an indispensable part of the non-equilibrium driving force contributes directly to the non-equilibrium dissipation in terms of the entropy production rate. The free energy dissipation computed in this way has been successfully used to cost-performance trade-off in biological systems whose energy sources are ATP, GTP and SAM [26, 27, 43]. In our present work, the energy cost is not explicit but implicitly represented by the entropy production. In the more elaborate models including explicit metabolic consumptions, the flux and therefore the corresponding entropy production rate is directly related to the metabolic energy consumption in analogy to ATP pump as the input to the biochemical system [26, 27, 40, 43].

### Biophysical circuit model for working memory

Here we explored a simplified biophysics-based model that can account for the working memory function [13, 35–37]. The model is comprised of two selective, excitatory populations, labeled 1 and 2. These two populations have self-excitations from the strong recurrent excitatory connections that are dominated by NMDA-mediated receptors and inhibit each other through a common pool of inhibitory interneurons. With a mean-field approach, the firing-rate dynamics of each excitatory population can be described by the dynamics of the average NMDA synaptic gating variable *S*_*i*_. This approximation is based on the fact that the synaptic gating variable of NMDA receptors has a much longer decay time constant than other timescales in the system, whereas the other synaptic variables instantaneously reach their steady state. The mutual inhibition can also be linearized so that the projections between the two excitatory populations are effectively represented by negative weights. Therefore, the dynamics of the working memory circuit model can be described as follows: $\frac{dS_{i}}{dt}=-\frac{S_{i}}{\tau_{S}}+\left(1-S_{i}\right)\gamma f\left(I_{i,tot}\right),i=1,2$, where firing rate *r*_*i*_ of neural population *i* is a function of total input current *I*_*i*,*tot*_ that can be written as [35, 37]: $r_{i}=f\left(I_{i,tot}\right)=\frac{aI_{i,tot}-b}{1-exp[-d\left(aI_{i,tot}-b\right)]},i=1,2$. The corresponding parameters are *a* = 270(*VnC*)^−1^, *b* = 108*Hz*, *d* = 0.154*s*, *γ* = 60*ms* and *τ*_*S*_ = 100*ms*. The total synaptic input currents of the two neural populations dominated by the NMDA receptors are: *I*_1,*tot*_ = *J*_11_ *S*_1_ − *J*_12_ *S*_2_ + *I*_0_ + *I*_1,*ext*_, *I*_2,*tot*_ = *J*_22_ *S*_2_ − *J*_21_ *S*_1_ + *I*_0_ + *I*_2,*ext*_. Here the NMDA synaptic couplings are *J*_11_ = *J*_22_ = 0.30*nA*, *J*_12_ = *J*_21_ = 0.05*nA*, and *I*_0_ = 0.31*nA* is the average background synaptic input. *I*_1,*ext*_ and *I*_2,*ext*_ are external inputs to the two selective populations, respectively. The particular values of the parameters in the model here are the same as the ones shown in the previous work [13, 35].

To characterize WM in this circuit model, we explored the generation of stimulus-selective persistent activity states and their robustness against random fluctuations and distractors. In each trial, the random fluctuation is introduced by an uncorrelated standard Gaussian noise term whose amplitude measured by the diffusion coefficient **D** is set as *D* = 1.4*10^−5^. During the loading phase, the target stimulus is simulated as a current applied to population 1, whose amplitude is 0.02*nA*. During the maintenance phase, there are no external currents specifically applied to both selective populations in the scenario without distractors. If a distractor is needed to be considered, it is stimulated as a current applied to population 2. The amplitude of the distractor-related current is also set as 0.02*nA*, equal to the target one.

Furthermore, we quantitatively uncovered non-equilibrium potential landscapes from the underlying dynamics to explore the global properties of working memory neural networks. The global robustness of the neural circuits can be explored by quantifying the probability landscape topography and the kinetics of state switchings. The activity (firing rate)*r*_*i*_ of selective excitatory population *i* is a monotonically increasing function of the corresponding average gating variable *S*_*i*_. This implies that a larger *S*_*i*_ will lead to higher activities of neural population *i*. Therefore, quantifying the landscape of the network in the state plane of (*S*_1_, *S*_2_) is better for global understanding the dynamical process of working memory. We can write down the corresponding Langevin equations as: $\frac{dI_{i,tot}}{dt}=F\left(I_{1,tot},I_{2,tot}\right)+\zeta$ through the transformation of coordinates(**S** to **I**) because the noise term *ζ* is actually added to driving force of the total current of neural population *i*. Then, we can obtain the potential landscape as the function of *S*_1_ and *S*_2_ with the method we discussed above through the transformation of coordinates(**I** to **S**).

## Results

### The working memory function in the attractor landscape framework

As we have introduced, working memory(WM) is associated with the persistent activities of the neurons in the prefrontal cortex of the brain during a mnemonic delay [11, 13, 14]. However, the realistic neural networks are inherently noisy, e.g. uncorrelated noises due to stochastic inputs from different neurons and finite numbers of molecules in individual neurons and synapses. Moreover, the system may be influenced by the external distractor stimuli. A WM circuit with an emphasis on robustness requires shielding these internal noises and external distractions. Previous studies suggested a qualitative concept of attractor dynamics to describe the stability of the memory states [13, 35]. For example, if learning has not been strong enough, the network may leave the given selective attractor due to the fluctuations. In addition, a new stimulus can destabilize the present attractor, which may drive the network to another attractor corresponding to the incoming stimulus. Nevertheless, the WM function in the attractor landscape framework still needs to be quantified in a a universal way. Applying a general non-equilibrium landscape and flux approach(see details in the Method section) to a biophysically based WM model, we explicitly quantified the underlying landscape performing the WM function.

**Fig 1:**
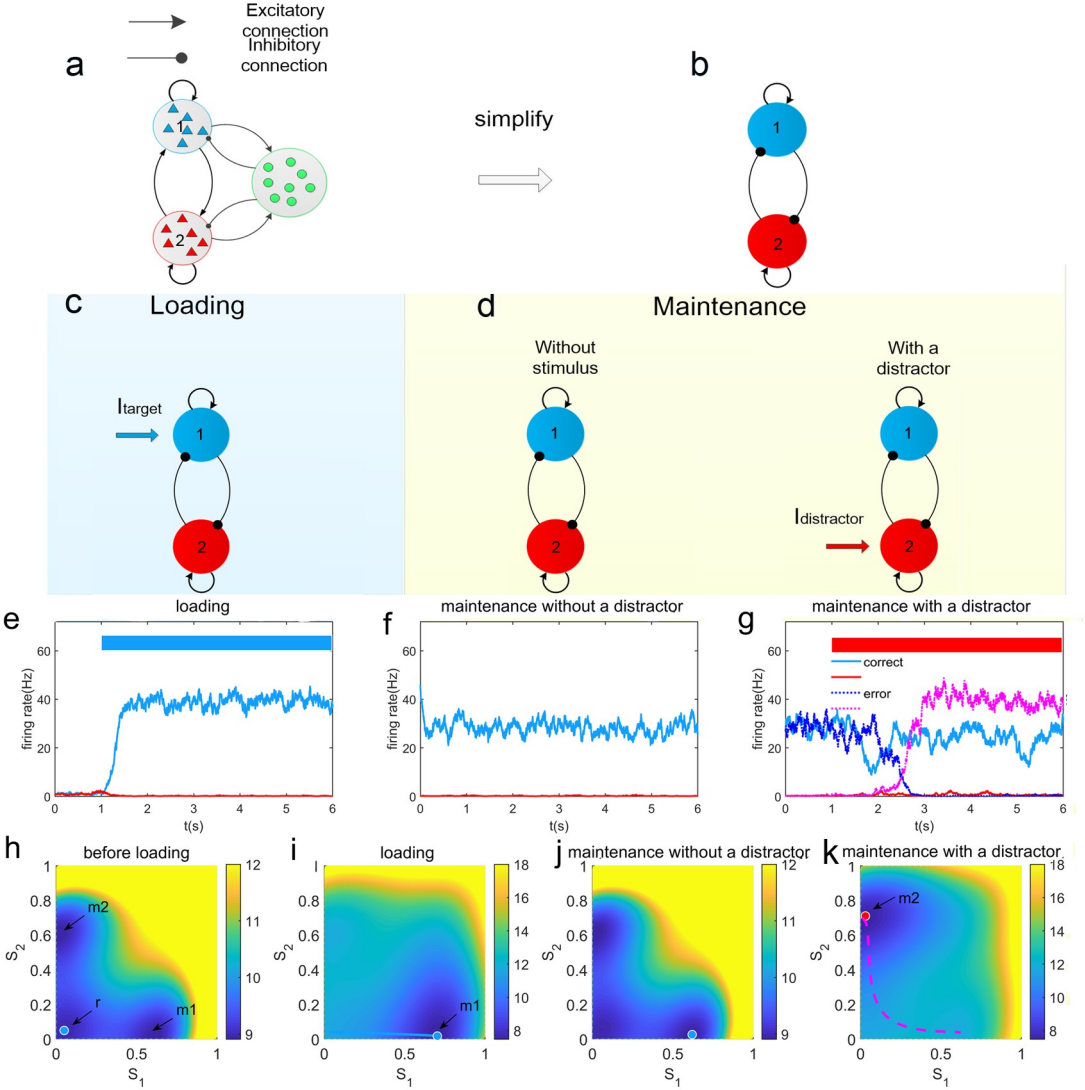
The schematic diagram of the circuit model for working memory. (a)The model is comprised of two selective, excitatory populations, labeled 1 and 2. Each excitatory population is recurrently connected and inhibit each other through a common pool of inhibitory interneurons. (b)The circuit model can be simplified through a mean field approach and the linearization of the inhibition. The effective inhibition from the pool of interneurons can be implicitly represented by the inhibitory connections between the excitatory populations. (c-d)The schematic diagrams for the WM during different phases in a WM task. During the loading phase, a target stimulus is presented. During the maintenance phase, this target should be held in WM against random fluctuations and distractors after the stimulus offset. (e-g)Neural activities of single trails during different phases. The colored bars mark the presentation of the stimulus input to the population 1(blue) and 2 (red), as the target and distractor stimulus, respectively. The blue and red lines represent the activities of population 1 and population 2, respectively. (e)Before the stimulus onset, the system stays at the resting state with both populations 1 and 2 at low activity. A target stimulus can quickly induce a stimulus-selective, high activity state. (f)This stimulus-selective state can be maintained in the absence of the target stimulus. (g)Correct and error trails in the presentation of a distractor stimulus. (h-k) The corresponding potential landscapes in the (*S*1, *S*2) state space during different phases. The dimensionless quantities *S*1 and *S*2 are average synaptic gating variables of the two selective populations, which can represent the mean population activities. The label *r* indicates the attractor representing the resting state. The attractors representing the target-related and distractor-related memory state are labeled with *m*1 and *m*2, respectively. After the onset of the target stimulus, the target-related memory state becomes dominated. The blue solid line indicates the transition from the resting state to the target-related memory state. (k)During the maintenance phase, the underlying potential landscape is changed by the presentation of a distractor stimulus, which may induce the transition to the distractor-related attractor(pink dashed line). In this figure, we used the baseline values of circuit connection strengths as *J*_+_ = 0.30*nA*, *J*_−_ = 0.05*nA*.

In general, positive feedback can work as the basic principle for generating persistent states(attractors). Here we use a reduced version of the spiking neuronal network models comprised of integrate-and-fire types through a mean-field approach [11, 13, 35], which can reproduce most of the psychophysical and physiological results in delayed response tasks [35, 45, 46]. In this model, stimulus-selective persistent activity can be maintained through the combined effects of recurrent excitation, mediated by the slow NMDA receptors, within a selective population and mutual inhibition, mediated by the GABAergic interneurons, between the populations(Fig 1(a) and 1(b)).

Fig 1(c) and 1(d) show the schematic diagram of the circuit model during different phases in a WM task. In Fig 1(e)–1(j), the dynamics of the circuit are described by the representative trials(the time course of the firing rate in each population) and the underlying potential landscapes(*U* = −*lnP*_*ss*_), which depend on the phase of the task. The corresponding potential landscapes are shown in the (*S*1, *S*2) state space, where the average synaptic gating variables *S*1,*S*2 are dimensionless quantities that can indicate the average activities of the two populations. In the absence of a stimulus, the system can hold three attractors: one corresponds to the symmetric resting state and the other two for the two asymmetric memory states(Fig 1(h)). Before the onset of the target stimulus, the system stays at the resting state where both populations are at low activities. Once a target stimulus is presented(a current *I*_*target*_ applied to population 1), the memory attractor that corresponds to the state of the target-selective population being sufficiently activated becomes dominated, while the resting state disappears(Fig 1(i)). State transitions from the resting state to the memory state can occur if the stimulus is strong enough. After the stimulus offset, the system can be maintained in the target-related memory attractor(Fig 1(j)). During the maintenance phase, a distractor stimulus(an input to a different population other than the target-selective one) can change the topography of the underlying landscape(Fig 1(k)). The distractor-related memory attractor becomes dominated and both the resting state and the target-related memory state become less stable. The target-related persistent activity can be either distracted(dashed lines in the Fig 1(g) and 1(k)), which means the failure of maintaining a stable memory state, or not being distracted(solid lines in the Fig 1(g)). The robustness of WM state against random fluctuations and external distractors depends on the network structure.

#### The robustness in a “remember the first” working memory task

Working memory(WM), as the ability to temporarily maintain and manipulate information, is a cornerstone of many cognitive functions. A WM circuit should also switch its operating mode according to different functional demands. In a “remember the first” WM task, the WM circuit needs to store the initial stimulus while filtering out noises and subsequent stimuli. At this point, WM robustness is emphasized. Since the positive feedback(self-excitation and mutual inhibition) serves as the mechanism of generating mnemonic persistent activity [19], we investigated how excitation and inhibition implemented by NMDA and GABA synapses affect the stability of the persistent states under the landscape framework.

**Fig 2:**
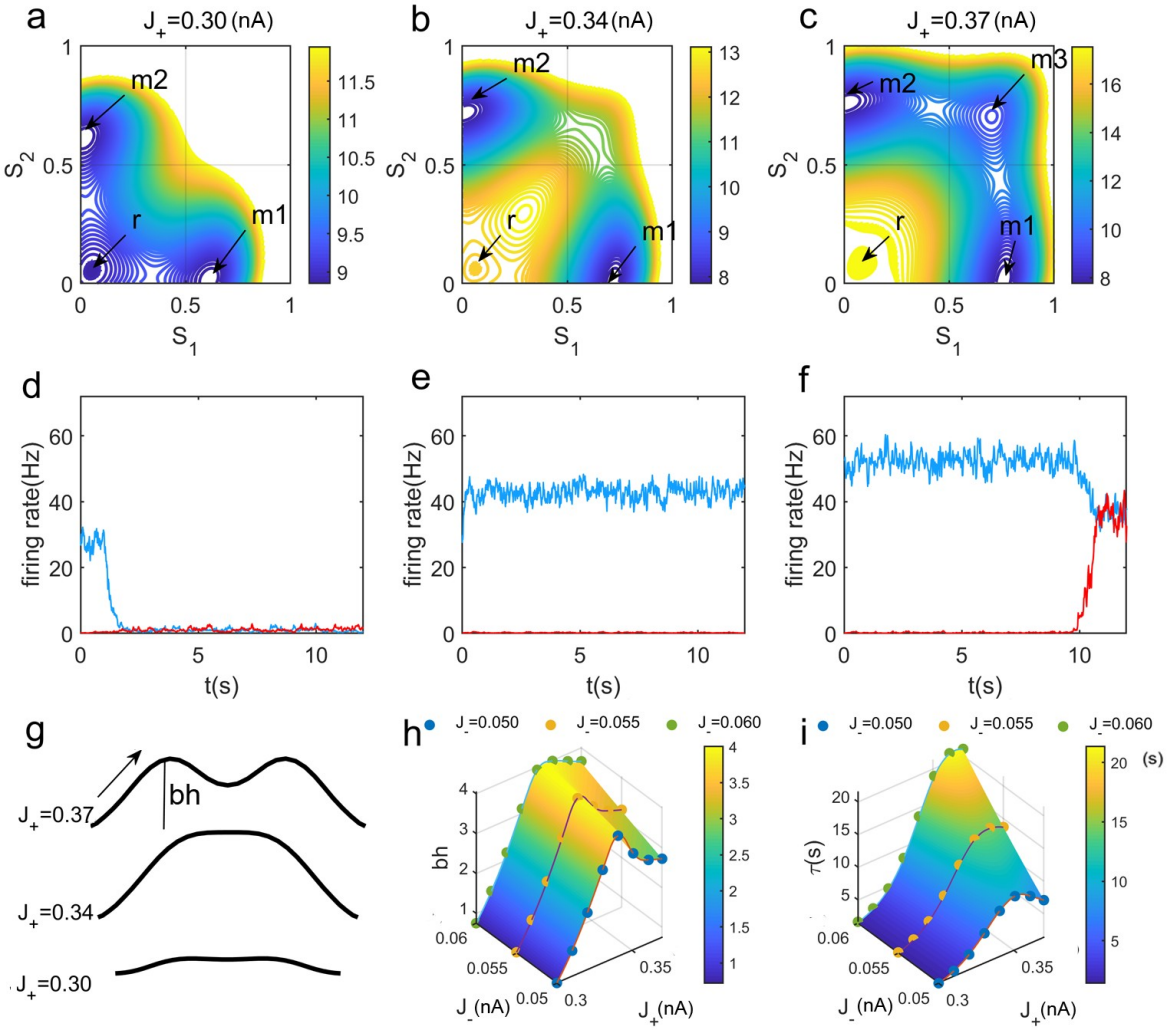
The robustness against random fluctuations during the maintenance phase. (a-c) The potential landscapes for different self-excitations *J*_+_ = 0.30, 0.34, 0.37*nA* after the stimulus offset. Here *J*_−_ = 0.05*nA* in (a-g). The attractor *r* for the resting-state becomes weaker while the attractors *m*1 and *m*2 for the two memory states become stronger with increasing *J*_+_. A new intermediate state *m*3 between the two memory state can emerge for further increasing *J*_+_ in Fig 2(c). (d-f)Neural activities of the single trails correspond to the potential landscapes for increasing *J*_+_ in the top panels. The blue and red lines represent the activities of population 1 and population 2, respectively. For both smaller and quite large *J*_+_, the system can be driven away from the target-related WM attractor under noise interferences. (g)The schematic diagram of the barrier heights on the corresponding potential landscapes for increasing *J*_+_. The barrier height is defined as the difference between the *U*_*min*_(the potential minimum of the present memory attractor) and the *U*_*saddle*_(the potential at the saddle point or barrier top between this memory attractor and its neighboring attractor). If the system tries to escape from the current memory state, the corresponding barrier needs to be crossed. (h-i)Robustness of WM against random fluctuations as a function of self-excitations *J*_+_ and mutual inhibition *J*_−_ through quantifying the corresponding barrier height and the mean first passage time(MFPT). The MFPT represents the average kinetic time of switching from the present attractor to a neighboring one. It measures the average transition time from the attractor *m*1 representing stimulus 1 to the resting state *r* before the emergence of the intermediate state for smaller self-excitation(*J*_+_ < 0.35*nA*). For the case that the intermediate state *m*3 emerges due to increasing self-excitation(*J*_+_ > 0.35*nA*), the MFPT measures the transition time from the attractor *m*1 to the intermediate state *m*3.

We first explored the scenario that there is no external distractor and the robustness of WM is influenced by the non-specific random fluctuations. The robustness against random fluctuations is determined by both the magnitude of fluctuations and the network structure. It is expected that the larger magnitude of the random fluctuations leads to weaker robustness. Therefore, the present work focuses on the role of the network structure on the robustness with the same magnitude of the fluctuations. Fig 2(a)–2(c) show the underlying potential landscapes(*U* = −*lnP*_*ss*_) of the WM circuit during the maintenance phase for different strengths of self-excitation *J*_+_. The corresponding neural activities for the representative single WM trials are shown in Fig 2(d)–2(f). We found that as *J*_+_ increases, the attractors representing the memory states on both sides become deeper (or stronger) while the central attractor representing the resting state becomes shallower (or weaker). This leads to the fact that the noise cannot easily move the system from the memory attractor to the resting one(Fig 2e). However, it is notable that a new intermediate state with both populations being activated emerges when the self-excitation gets even stronger(Fig 2c). Such an intermediate state reduces the barrier between the two memory attractors. As a result, the transitions are more likely to occur. A benefit of the landscape approach is that we can quantify the stability of each attractor through the heights of the separating barriers.

Fig 2g shows the schematic figure of how the barrier heights change with different *J*+ on the underlying potential landscapes(*U* = −*lnP*_*ss*_). A WM attractor with higher barrier height is more robust to noises. In addition to recurrent excitation, positive feedback can also arise from the mutual inhibition [47, 48]. In Fig 2h, we show more details on the crucial role of the circuit structure(both self-excitation and mutual inhibition) in determining the robustness against random fluctuations in terms of the barrier height. We can see that the changes in barrier height for a memory state with increasing self-excitation *J*_+_ is not monotonous. It increases first, and then decreases after the emergence of the intermediate state. However, stronger mutual inhibition(*J*_−_) can monotonically induce better robustness by means of supporting higher separating barriers.

Besides the depth of the basins of the attractions, the overall stability of the different attractors is also affected by the breadth and the distance apart of the basins. To further characterize the robustness in WM, we quantified the mean first passage time(MFPT) [38], which represents the average kinetic time of switching from the present attractor to a neighboring one, in addition to the corresponding barrier heights, for different network structure conditions. After the target stimulus offset, the system is disturbed by random fluctuations. As shown in Fig 2i, the MFPT from the target-related memory attractor to its neighboring attractor is closely related to the corresponding barrier height. It will take a longer time to escape from the present attractor with a higher barrier.

**Fig 3:**
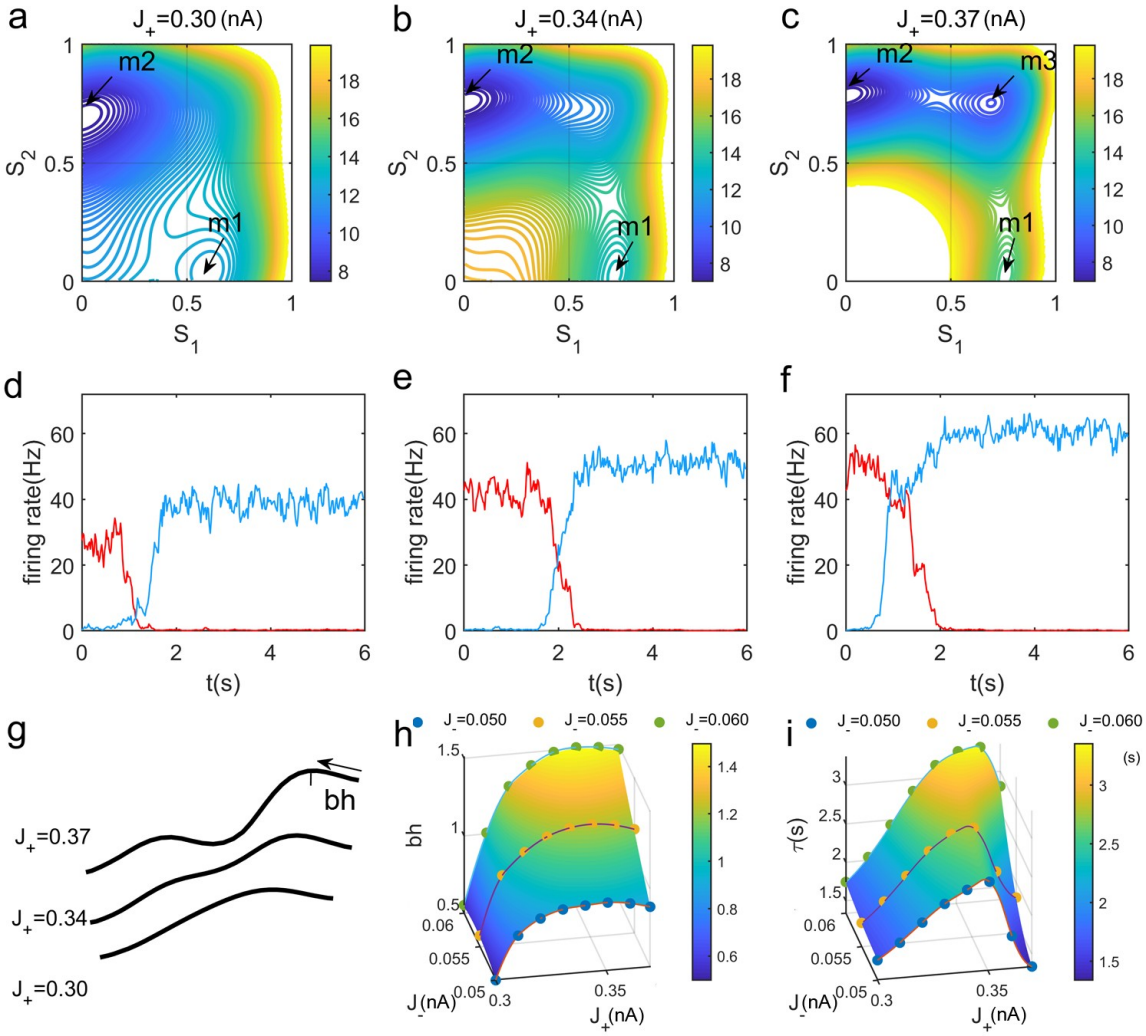
The robustness against distractors during the maintenance phase. (a-c) The potential landscapes for different self-excitations *J*_+_ = 0.30, 0.34, 0.37*nA* in the presence of a distractor stimulus. Here *J*_−_ = 0.05*nA* in (a-g). The attractor *r* for the resting-state cannot be held and the distractor-related memory attractor *m*2 becomes dominated. There is also a new intermediate state *m*3 between the two memory states for further increasing *J*_+_ in Fig 3(c). (d-f)Neural activities of the single trails correspond to the potential landscapes for increasing *J*_+_ in the top panels. The blue and red lines represent the activities of population 1 and population 2, respectively. The system can be driven away from the target-related WM attractor by the intervening distractor. (g)The schematic diagram of the barrier heights on the corresponding potential landscapes for increasing *J*_+_. (h-i)Robustness of WM against distractors as a function of self-excitations *J*_+_ and mutual inhibition *J*_−_ through quantifying the corresponding barrier height and the mean first passage time(MFPT).

Furthermore, we explored how the network structure influences the robustness against distractors. Here a distractor stimulus is defined as an input applied to the other selective population different from the one receiving the target stimulus. The amplitudes of the distractor stimulus and the target stimulus are set equal. As shown in Fig 3(a)–3(c), the distractor stimulus breaks the symmetric landscape and the distractor-related memory state becomes dominant. Such a distractor signal can erase the previous memory and further encode the new one. We found the similar results to the ones in the absence of a stimulus that are shown in Fig 2. The stronger mutual inhibition can enhance the robustness against distractions. However, the effects of increasing self-excitation are not monotonous. The robustness against distractors becomes better until the intermediate state emerges. It appears that the intermediate state reduces the difficulty of jumping from the present memory attractor to the new one through lowing the corresponding barriers. The robustness is the best in an intermediate range of *J*_+_ with larger *J*_−_.

We have shown that modulations of the self-excitation *J*+ and mutual inhibition *J*− in the WM circuit can have different influences on the robustness of WM state to random noises and distractors. We then asked the question whether the WM robustness can be enhanced through a concerted modulation in both *J*+ and *J*−. To quantitatively address this issue, we investigated the implications of dopamine *D*_1_ receptor activation on WM functions, which can simultaneously influence the self-excitation and mutual inhibition strengths through modulating the NMDA conductances on the stimulus-selective neurons in the WM circuit. Since the coefficients *J*+ /*J*− represent the mean effective coupling strengths within each selective population/between the two selective populations which are mediated by NMDA receptors [35], the modulation of different levels of *D*_1_ activation on the strengths of self-excitation *J*+ and mutual inhibition *J*− can be specifically modeled as these effective coupling coefficients being multiplied by a sigmoid function *Ce*(1 + 0.2/(1 + *exp*((0.8−*D*_1_)/0.25))). Here the variable *D*_1_ represents the relative change of the simulated dopamine *D*_1_ receptor activation, and *Ce* is chosen so that the factor is equal to 1 when *D*_1_ = 1. This follows the previous theoretical work about the differential dopamine *D*_1_ modulation of NMDA conductances in a cortical network model of WM [11]. We found that both self-excitation and mutual inhibition increase at higher *D*_1_ activation levels(S1(a) Fig). The resulting robustness against both random fluctuations and distractors are greatly enhanced(S1(b) and S1(c) Fig).

#### The relationship between the robustness and flexibility in a “remember the last” working memory task

In our daily lives, our minds may often flit from thoughts to thoughts, which indicates that the dynamics of the underlying neural circuits can be highly flexible upon the behavior task demands [10, 24]. For example, in some cognitive tasks the most recent stimulus is needed to be stored. This requires the WM circuit operating in a “remember the last” mode and the flexibility to the new stimulus is emphasized. Whereas we have explored the network structure conditions for increasing the robustness of the memory representation in the “remember the first” tasks, it is natural to think that they have the opposite implications on the flexibility to switch between dynamical states. Indeed, for lower self-excitation and mutual inhibition, the system is more flexible while less robust against both random fluctuations and distractors/new stimuli(Figs 2(h), 2(i), 3(h) and 3(i)). However, it is notable that the flexibility to a new stimulus does not have to be increased at the expense of robustness to all perturbations. In fact, a WM circuit during the maintenance phase in a “remember the last” task should be robust against random fluctuations before the onset of a new stimulus. This is because that if there is no new stimulus presented, the current memory state is the correct one that should be maintained robustly. This then raises the question what the network mechanism is for such flexibility in the “remember the last” task and whether a single WM module can reconfigure their dynamical properties, switching between different operating modes.

**Fig 4:**
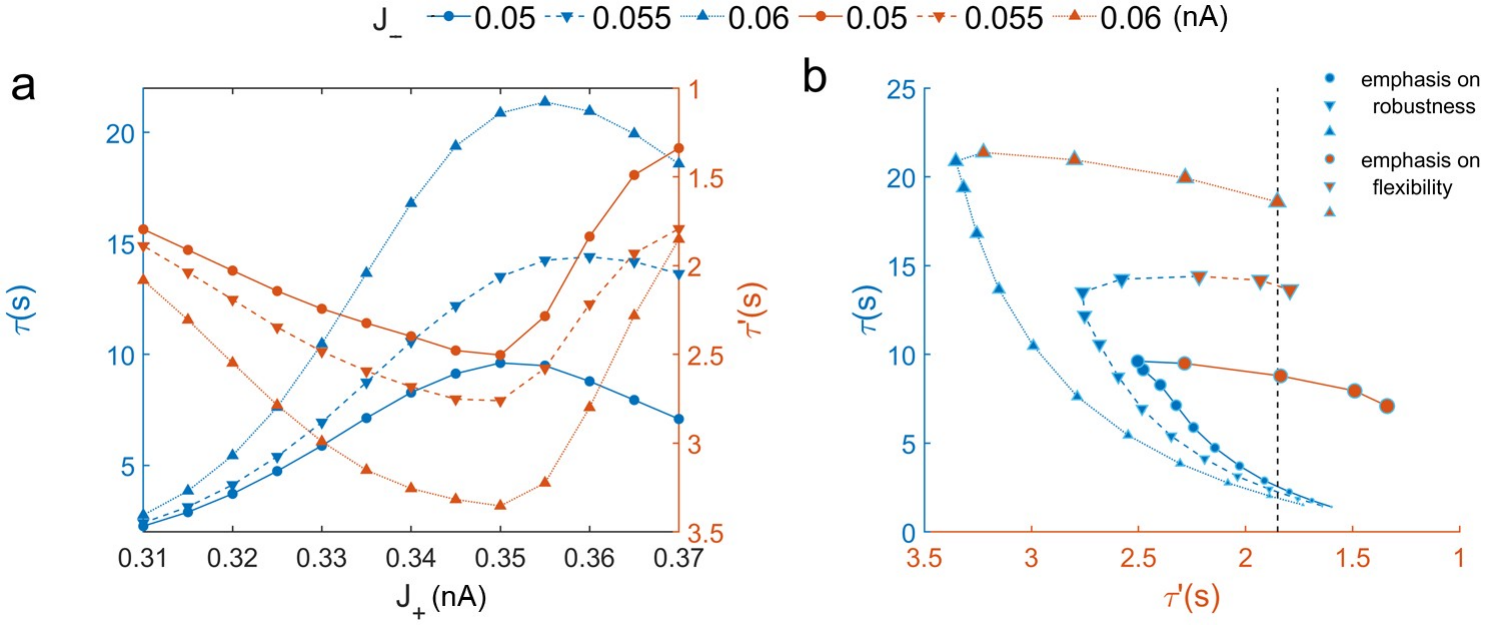
The relationship between the robustness against random fluctuations and the flexibility to a new stimulus. (a)The robustness against random fluctuations and flexibility to a new stimulus as functions of self-excitation *J*_+_ and mutual inhibition *J*_−_. The curves marked with the circles, lower and upper triangles represent different mutual inhibition *J*_−_ = 0.05, 0.055, 0.06*nA*, respectively. *τ* and *τ*′ represent the MFPT in the absence and presence of a new stimulus respectively. Larger *τ* indicates better robustness against random fluctuations and smaller *τ*′ implies better flexibility to a new stimulus. (b)The relationship between the robustness and flexibility. The larger size of markers on each curve indicates stronger self-excitation *J*_+_. The emergence of the intermediate state(the inflection point on each curve) leads to better flexibility to a new stimulus, while the robustness against random noise might be not significantly reduced. The blue part of each curve represents that the system is in the configuration of emphasizing the robustness where strengthening the self-excitation *J*_+_ enhanced the robustness against random fluctuations at the expense of reducing the flexibility to a new stimulus. The red part of each curve implies that the flexibility to a new stimulus can be enhanced without seriously reducing the robustness against the random fluctuations through strengthening *J*_+_.

Instead of a simple trade-off between robustness and flexibility to ongoing uncorrelated noise in the scenario without external stimulus, we found the enhanced flexibility to a new stimulus does not conflict with the good robustness against random fluctuations. This can be achieved through the emergence of the new intermediate state due to the increased self-excitation, which can significantly enhance the flexibility to a new stimulus without seriously reducing the robustness to random fluctuations. This result is more explicitly shown in Fig 4(a) and 4(b), where larger *τ* indicates better robustness against random fluctuations and smaller *τ*′ implies better flexibility to a new stimulus. The reversal of the positive relationship between the robustness(*τ*) and self-excitation *J*_+_ on each curves is a result of the emergence of the intermediate state. Better robustness against the random fluctuations(worse flexibility to a new stimulus) can always emerge from the enhanced mutual inhibition *J*_−_. For robustness, at the same *J*_+_, the blue upper triangle marked line is always higher than the lower triangle line and the lower triangle line is always higher than the circle marked line. Vice versa for the flexibility (red lines).

For the same flexibility to a new stimulus measured by *τ*′(dashed line in Fig 4(b)), the circuit with both stronger self-excitation *J*_+_ and mutual inhibition *J*_−_ has a better chance of modest reduction of robustness against random noise with significant increase of the flexibility(see the almost horizontal red part of curves marked with the upper and lower triangles in Fig 4(b)). This is in contrast to the case where the flexibility to a new stimulus can only be enhanced by the expense of reducing the robustness against the random fluctuations at lower self-excitation and mutual inhibition(the blue part of each curve). Therefore, robustness is not always simply against flexibility. Our results suggest that there is an optimal compromise between robustness and flexibility for higher self-excitation and mutual inhibition.

### The energy cost enhances the performance in working memory

Having characterized the role of the network architecture in working memory, we further explored the underlying physical mechanisms. Notably, the persistent activities in a working memory circuit are not stable states at equilibrium conditions. Neural networks are open systems having constant exchange of material, information and energy with the environment. Therefore, they are intrinsically non-equilibrium. The main characteristic of such non-equilibrium system is that the driving force is determined by both the gradient of the non-equilibrium landscape and the steady-state probability flux [29, 30]. The steady-state probability flux, which satisfies the local probability conservation law: ∂*P*(**x**, *t*)/∂*t* = −∇⋅**J**, can be non-zero when ∂*P*(**x**, *t*)/∂*t* = 0. Therefore, the non-zero probability flux representing the net input and output of the system breaks the detailed balance and describes the degree of the the system away from the equilibrium(see more details in the Method section). Furthermore, being different from the equilibrium cases, the persistent activities in non-equilibrium systems are maintained by the energy consumption. The thermodynamics cost for maintaining the steady state in non-equilibrium systems can be characterized by the corresponding entropy production rate(*epr* = ∫*d* **x**(**J**⋅**D**^−**1**^⋅**J**)/*P*), which is directly related to the flux [29, 34]. Here **D** is the diffusion coefficient giving the magnitude of the fluctuations.

**Fig 5:**
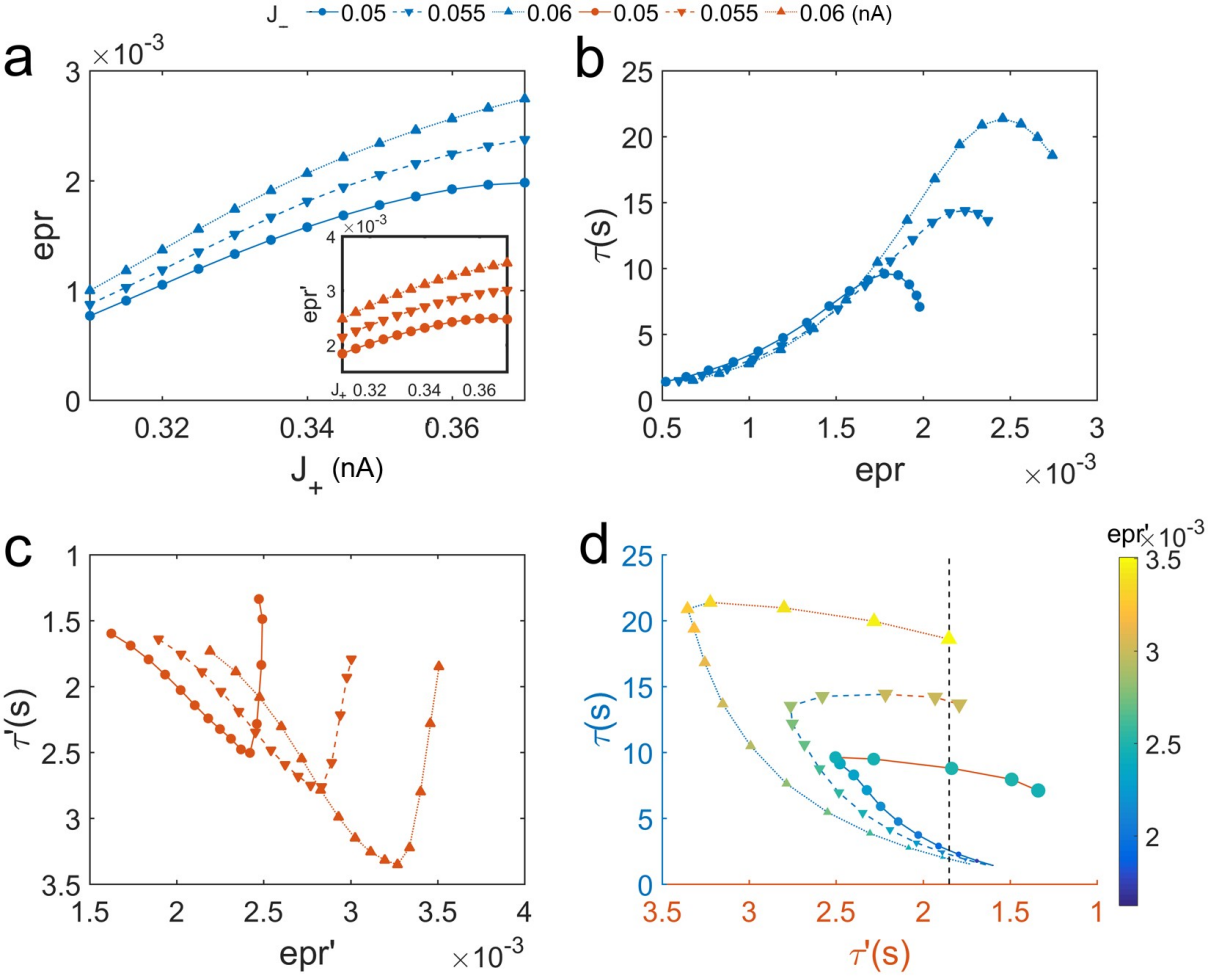
The energy cost quantified by the entropy production rate in working memory. (a)The entropy production rate increases with increasing *J*_+_ and *J*_−_ in the absence and presence of a new stimulus. The curves marked with the circles, lower and upper triangles represent different mutual inhibition *J*_−_ = 0.05, 0.055, 0.06*nA*, respectively. (b-d)The relationship among the robustness against random fluctuations, flexibility to a new stimulus and the entropy production rate. (b)Before the emergence of the intermediate state(before the inflection point on each curve), better robustness against the random fluctuations quantified in terms of the MFPT *τ* requires more energy cost. (c)After the emergence of the intermediate state, the additional energy is used for supporting the new attractor which can enhance the flexibility to a new stimulus quantified in terms of the MFPT *τ*′. The colors of circles in (d) indicate the corresponding entropy production rate. The larger size of markers on each curve indicates stronger self-excitation *J*_+_.

Recent studies show that there is a trade-off between the performance/functional robustness and energy dissipation in the biological systems [26, 27, 32, 34]. To investigate whether the performance in working memory can be enhanced through the energy expenditure, we quantified the entropy production rate(epr) for varying strengths of neural interactions in the circuit(Fig 5(a)–5(c)). We can first consider the scenario that the WM circuit emphasizes on the robustness in “remember the first” tasks. In Fig 5(a), we can see both strengthened self-excitation and mutual inhibition will lead to more energy cost, irrespect to the presence of external stimulus or not. Fig 5(b) shows that the robustness against the random fluctuations first arises with an increase in epr due to the increasing *J*_+_. This positive relation reverses after the emergence of the intermediate state as *J*_+_ increases further. In addition, the better robustness induced by increasing *J*_−_ is accompanied by larger epr. In the presence of distractors, we can find similar relationship between the robustness(vice versa for the flexibility)and the epr(Fig 5(c)). Our results suggest that in the case of low circuit connections with less energy cost, the system is more flexible while less robust to both random fluctuations and distractors. The circuit is capable of maintaining robust mnemonic persistent activity only when more energy is supplied. Notably, the emergence of the new intermediate state increases the energy cost while adversely affects the robustness performance in a “remember the first” task. However, it is beneficial to the performance of the WM tasks that emphasize on the flexibility to a new stimulus.

**Fig 6:**
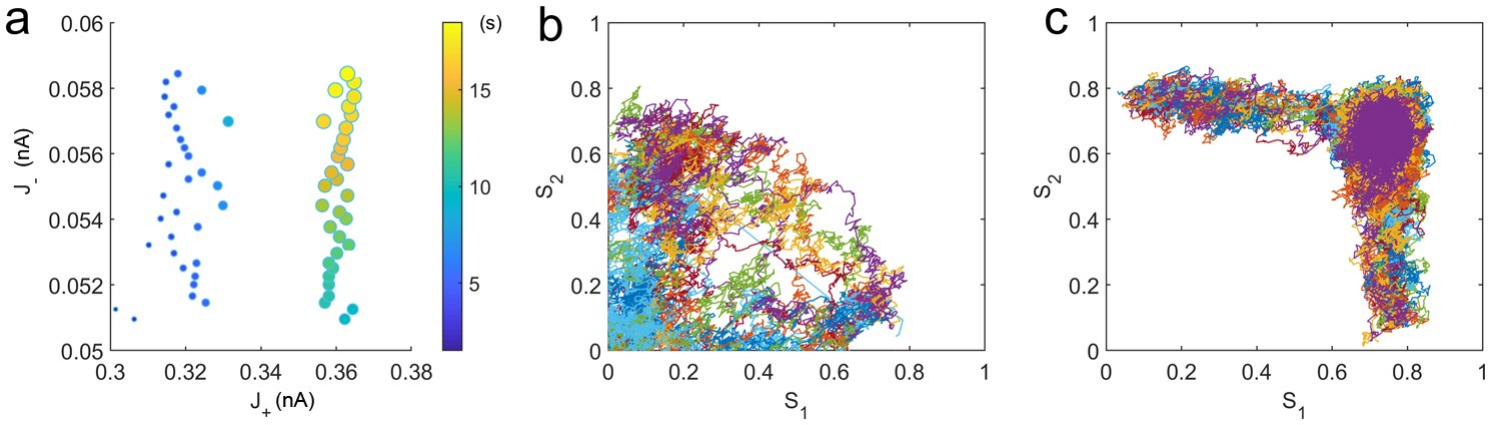
The emergence of the intermediate state can improve the performance in a WM task with an emphasis on flexibility to a new stimulus. (a)Better robustness against random fluctuations with the same flexibility to a new stimulus due to the emergence of the intermediate state. For any specific mutual inhibition *J*_−_, we choose a pair of self-excitation *J*_+_, which correspond to the same flexibility to a new stimulus from the data shown in Fig 4(a). Each pair of *J*_+_ corresponds to different robustness to random fluctuations. The circles on the right side indicate the robustness against random fluctuations in terms of both size and color(the escape time from the original memory attractor) for systems in the presence of the intermediate state. The circles on the left side indicate the robustness for systems without the intermediate state. For any specific pair of *J*_+_ with the same *J*_−_ and also the same flexibility to new signals, the robustness against random fluctuations is better for the system with the presence of the intermediate state(circles on the right side). (b-c) The distributions of the single trials switching from the target-related memory state to the distractor-related one in the absence and presence of the new intermediate state. It is obvious that the pathways are more restrained with the intermediate state. The self-excitation *J*_+_ = 0.30, 0.36*nA*, respectively with the mutual inhibition *J*_−_ = 0.05*nA*.

We have discussed that there is an optimal compromise between the robustness against the random fluctuations and the flexibility to a new stimulus for better performance in the “remember the last” WM tasks. To explore the cost for achieving such good performance, we show the relationship among the robustness, the flexibility and the energy cost in Fig 5(d). The sizes of the markers on each curves indicate the strengths of *J*_+_ while the colors of markers indicate the corresponding epr. We can see that the robustness against the random fluctuations always increases at the cost of flexibility to a new stimulus and the energy consumption at first, then the flexibility can be regained with a reduction in robustness and further increased energy cost after the new intermediate state emerges. For the curve marked with circles(the smallest *J*_−_ and the least energy cost), the enhanced flexibility is accompanied by an obvious reduction in the robustness. However, the two curves marked with upper and lower triangles, respectively(larger *J*_−_ and more energy cost) show that the good flexibility to a new stimulus can be achieved without significantly reducing the robustness against random fluctuations. This good performance in “remember the last” WM tasks enhanced by both increasing self-excitation and mutual inhibition is supported by larger energy consumption. Notably, the key element of the mechanism for achieving good performance in WM is the emergence of the new intermediate state. Although an additional energy is needed to support this intermediate state, better robustness against random fluctuations with the same flexibility to the new stimulus can be achieved due to the emergence of the intermediate state(Figs 5(d) and 6(a)).

## Discussion

Selective delay activity in cortex has been observed in various working memory tasks [14, 49, 50]. Such activity patterns can be sustained by positive feedback processes in a neural circuit. However, the brain is inherently noisy. Even with persistent states, noise may drive the system away from the original state and further make errors. The nature of a memory storage system indicates that the persistent states should be robust to noise. Until now, the precise mechanisms remain unclear. Previous theoretical research on network models of working memory applied the concept of attractor dynamics to describe the robustness in working memory [13, 17]. In the underlying attractor landscape or energy landscape, each persistent pattern has a basin of attraction, and the stability of each pattern can be represented by the depth of the basins and the distance apart from the basins. Nevertheless, such landscape is either not explicitly quantified [35, 51, 52] or only suitable for very specific circuits, e.g. the Hopfield circuit model with symmetric synaptic connections [2]. Instead of the explicitly quantified attractor landscape, previous modeling studies explored the robustness in working memory through following the corresponding neuronal activity trajectories in a limited time scale [13, 25]. The robustness investigated in this way can only reflect the local properties rather than the global natures such as the stability of other persistent states and the relative weights between them. Moreover, the accuracy of the results depends on the volume of the statistical data. Inspired from the thermodynamics and statistical mechanics, we developed a non-equilibrium potential landscape and flux framework for general neural circuits [28]. Applying this theoretical framework to a biophysically based circuit model that is able to perform working memory computations, we investigate how excitation and inhibition implemented by the NMDA and GABA synapses affect the network dynamics and further the robustness and flexibility in working memory.

Since we are interested in the global properties of working memory circuits in cognitive tasks based on the landscape framework rather than trying to account for details of every spiking neurons, we use a reduced version of the spiking neuronal network models comprised of integrate-and-fire types through a mean-field approach [11, 13, 35]. Although the simplified model is described by only two dynamical variables or effective populations, it captures the essential characters of PFC networks responsible for working memory: strong excitatory reverberation between spiking neurons, mediated by the slow NMDA receptors [53], and recurrent feedback inhibition, mediated by the GABAergic interneurons. Moreover, it can reproduce most of the psychophysical and physiological results in delayed response tasks [35, 45, 46].

In the present work, we investigated two kinds of noises that may induce the network to abandon a given selective attractor: the non-selective random noise and distractor stimuli. By quantifying the underlying landscape topography through the barrier heights and the mean transition time between different attractors, we found that the combined effects of both stronger recurrent excitation and inhibition can increase the robustness to each kind of noise in working memory. This result is consistent with previous studies showing that an increased activation of D1 receptors, which induces increases in both NMDA and GABA currents, is beneficial for the robust online maintenance of information [11, 17, 54]. Interestingly, our modeling study suggests that an intermediate state may emerge due to the further increase of self-excitation within selective neural populations. This intermediate state lowers the barrier between two memory attractors and significantly reduces the robustness against distractors. The enhanced mutual inhibition can delay the emergence of the intermediate state due to the increase of self-excitation. In S2 Fig, we show that the robustness against both the random fluctuations and the distractors are greatly enhanced for further increasing mutual inhibition *J*− in addition to the results shown in Figs 2 and 3. Therefore, our results suggest that strengthening the positive feedback in terms of increasing mutual inhibition is a better choice with an emphasis on the robustness against distractors.

Sometimes, the working memory system needs to emphasize on the flexibility to a new stimulus rather than the robustness against noises according to environmental conditions or behavioral task demands. Conventionally, it is thought that there is a conflict between the robustness and the flexibility in working memory [54]. However, emphasizing on the flexibility to a new stimulus does not mean that the dynamics of the system is not subject to any restrictions. For a “remember the last” WM task, the WM circuit during the maintenance phase should be robust against the random fluctuations before the onset of a new stimulus. Our results suggest that the emergence of the intermediate state due to the increase of self-excitation can enhance the flexibility to a new stimulus without significantly reducing the robustness to random fluctuations. We found there is an optimal range of network connections(“sweet spots”) for the trade-off between the robustness and flexibility in working memory. Such optimal configuration requires an appropriate combination of self-excitation and mutual inhibition. The self-excitation should be strong enough for supporting stable memory attractors and the additional intermediate state. Appropriate mutual inhibition is also necessary for ensuring the robustness to random fluctuations and also for preventing the intermediate state to become dominant.

Previous studies have investigated the biophysical mechanisms responsible for the tradeoff between the accuracy and flexibility in working memory, where the flexibility to switch between dynamical states is quantified in terms of the ability to erase memory(back to the resting state) using a negative input [25]. In the present work, we found that the transitions between different memory states can be promoted through an intermediate state in addition to the resting state. Comparing with the mechanism that switching the working memory system from one memory state to another through the resting state, the advantage of the mechanism in our model is that better robustness against the random fluctuations can be achieved with the same flexibility to a new stimulus. Fig 6(a) shows the robustness against the random fluctuations quantified by the escape time from the original memory attractor for two different mechanisms. In addition, the pathways of transitions from one memory state to another are more restrained with the presence of the intermediate state(see Fig 6(b) and 6(c)). This implies less uncertainty may happen for this mechanism.

The intermediate state in our model corresponds to high activities in both selective neural populations, and consistent experimental evidence has been found in delay response tasks [46]. Moreover, previous vivo recordings in the working memory experiments suggest that transitions between different activity patterns can occur, where the target stimulus-selective persistent activity pattern decreased and non-stimulus related activity slowly increased during the delay [17]. These experimental evidences provide support to the present mechanism in modulating the tradeoff between robustness and flexibility through the intermediate state during the delay. Predictions of our model can be tested in vivo by applying D1 receptor agonists and gradually blocking prefrontal cortex GABA receptors [55] in delayed reaction-type tasks.

Furthermore, we explored the physical nature underlying the biological function in the working memory circuit. Due to the non-equilibrium nature of neural circuits, the persistent activities in these systems are intrinsically non-equilibrium. The main characteristic of such non-equilibrium process is that the corresponding driving force for the dynamics is determined by both the gradient of potential landscape and curl flux, which is directly related to the energy cost for maintaining the non-equilibrium stable states in terms of entropy production rate. Our results suggest that the energy supply is crucial for the robustness of working memory. The enhanced flexibility to a new stimulus without greatly reducing the robustness to the random noises through increasing the mutual inhibitory connections in addition to increasing the self-excitatory connections is also supported by extra energy cost. In other words, the robustness against the random fluctuations is powered by energy dissipation, and more energy is needed to generate better flexibility to the new stimulus.

However, it is notable that the optimum performance(defined as good flexibility to a new stimulus without significantly reducing the robustness against random fluctuations) may not be achieved through the maximum energy cost. For a constant mutual inhibition, the excessively enhanced self-excitation may induce the overweighted intermediated state. This leads to that the system is easier to escape from the original memory state(losing robustness against the random fluctuations). With varying mutual inhibitions, the network with both greatly strengthened self-excitation and mutual inhibition(also larger energy cost) would bias towards better robustness against noises and distractors rather than the flexibility to a new stimulus.

**Fig 7:**
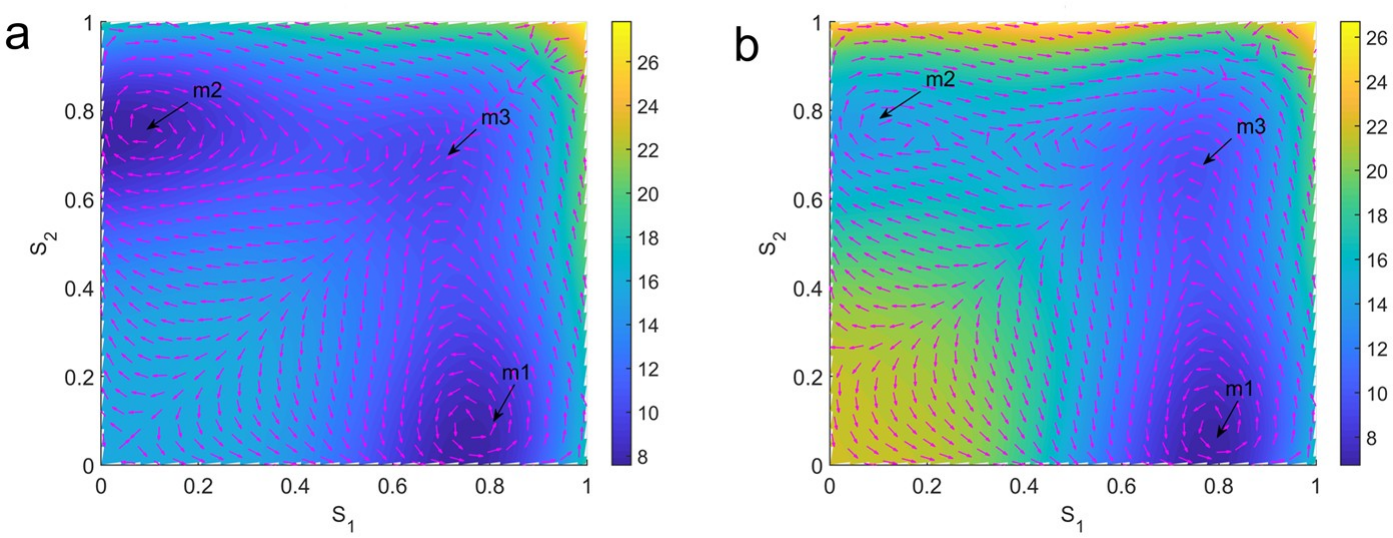
The non-equilibrium landscapes and flux in the absence and presence of a distractor stimulus. (a)There is no external stimulus presented. (b)A distractor stimulus is presented. The purple arrows indicate the flux part of driving force. *m*1 and *m*2 indicate the two memory attractors and *m*3 indicates the new intermediate state. Here *J*_+_ = 0.36*nA* and *J*_−_ = 0.05*nA*. The color map indicates the value of potential (*U* = −*lnP*_*ss*_) at each state, which is a dimensionless quantity.

Moreover, the non-equilibrium flux(indicated by the purple arrows in Fig 7) provides the main driving force for the transitions between different memory states against the gradient force from local attractor. In Fig 7, we can see that the flux drives the system away from the local attractor and further leads to another nearby attractor. Being different from the equilibrium cases, the non-equilibrium flux facilitates the biological functions in working memory from both the dynamical perspective and from the thermodynamic perspective(energy expenditure).

In conclusion, our non-equilibrium landscape and flux approach provides a general way to study the dynamics of neural circuits. In particular we investigate the working memory dynamics in terms of energy landscape, state transition and entropy production rate. The robustness of working memory can be quantified by the underlying landscape topography(barrier heights) and the corresponding mean transition time(MFPT) without the necessity of referring to the temporal dynamics of specific task. Therefore, the accuracy of the results can be less dependent on the volume of statistical data. Applying this approach, we uncovered that the emergence of the new intermediate state is the key element of the mechanism for achieving good performance in working memory. The advantage of the intermediate state mechanism is that it can provide better robustness against the random fluctuations with the same flexibility to a new stimulus. Being able to quantify the energy consumption in general physiological processes, our approach can facilitate the design of new network structure for cognitive functions with the optimal balance between performance and cost. Our work also provides a new paradigm for exploring the underlying mechanisms of many cognitive functions such as decision making and cognitive symptoms of schizophrenia from the landscape perspective(Growing evidence showing that deficits in working memory are prominent symptoms in schizophrenia) [56, 57].

## Supporting information

S1 Fig Dopaminergic neuromodulation on the WM function. (a)The modulation of different levels of *D*_1_ activation on the strengths of self-excitation *J*+ and mutual inhibition *J*−. The modulated self-excitation *J*+ and mutual inhibition *J*− can be specifically modeled as these effective coupling coefficients being multiplied by a sigmoid function *Ce*(1 + 0.2/(1 + *exp*((0.8 − *D*_1_)/0.25))). Here the variable *D*_1_ represents the relative change of the simulated dopamine *D*_1_ receptor activation, and *Ce* is chosen so that the factor is equal to 1 when *D*_1_ = 1. (b-c)The robustness against the random fluctuations and distractors in terms of the MFPT are enhanced for increased *J*+ and *J*− due to the larger *D*_1_ activation levels. The white asterisks indicate the modulated *J*+ and *J*−(the baseline *J*+ = 0.30 and *J*− = 0.05 multiplied by the sigmoid function *Ce*(1 + 0.2/(1 + *exp*((0.8 − *D*_1_)/0.25)))) and the corresponding MFPT.
(TIF)

S2 Fig The robustness against the random fluctuations and the distractors in terms of the MFPT for further increasing mutual inhibition *J*−. (a)The robustness against the random fluctuations and (b)The robustness against the distractors are greatly enhanced for further increasing mutual inhibition *J*−.
(TIF)
