# Supplementary figures and images for: Non-equilibrium landscape and flux reveal the stability-flexibility-energy tradeoff in working memory
Source: PLoS Comput Biol. 2020 Oct 2;16(10):e1008209. doi: 10.1371/journal.pcbi.1008209 (PMC7531819; doi:10.1371/journal.pcbi.1008209)

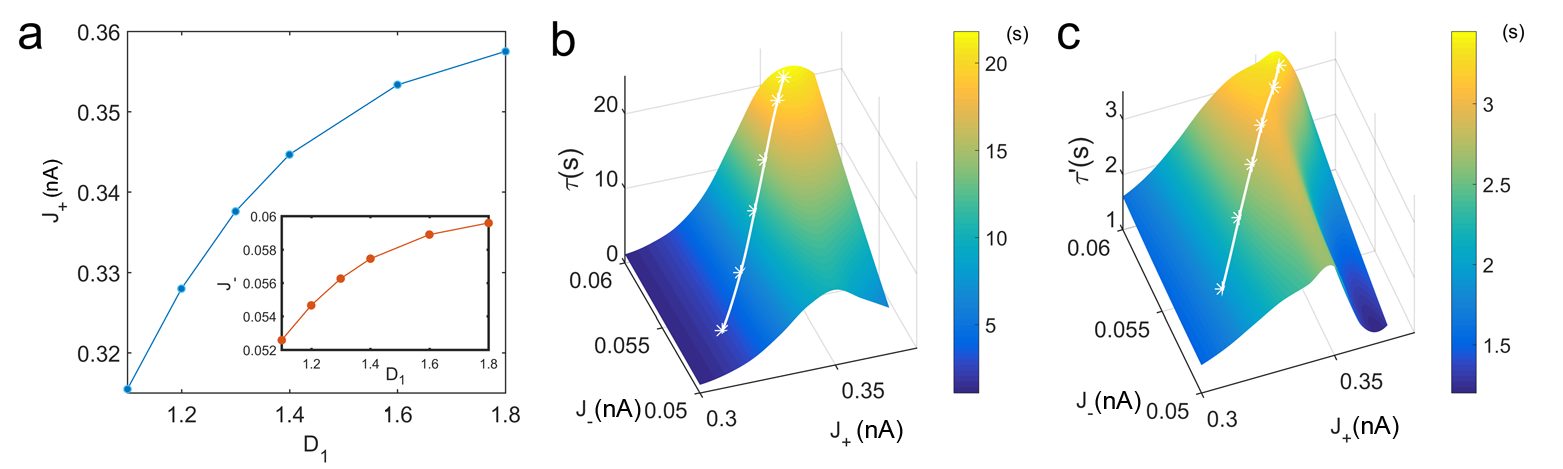

Supplement: S1 Fig — (a)The modulation of different levels of D1 activation on the strengths of self-excitation J+ and mutual inhibition J−. The modulated self-excitation J+ and mutual inhibition J− can be specifically modeled as these effective coupling coefficients being multiplied by a sigmoid function Ce(1 + 0.2/(1 + exp((0.8 − D1)/0.25))). Here the variable D1 represents the relative change of the simulated dopamine D1 receptor activation, and Ce is chosen so that the factor is equal to 1 when D1 = 1. (b-c)The robustness against the random fluctuations and distractors in terms of the MFPT are enhanced for increased J+ and J− due to the larger D1 activation levels. The white asterisks indicate the modulated J+ and J−(the baseline J+ = 0.30 and J− = 0.05 multiplied by the sigmoid function Ce(1 + 0.2/(1 + exp((0.8 − D1)/0.25)))) and the corresponding MFPT. (TIF) [file pcbi.1008209.s001.tif]

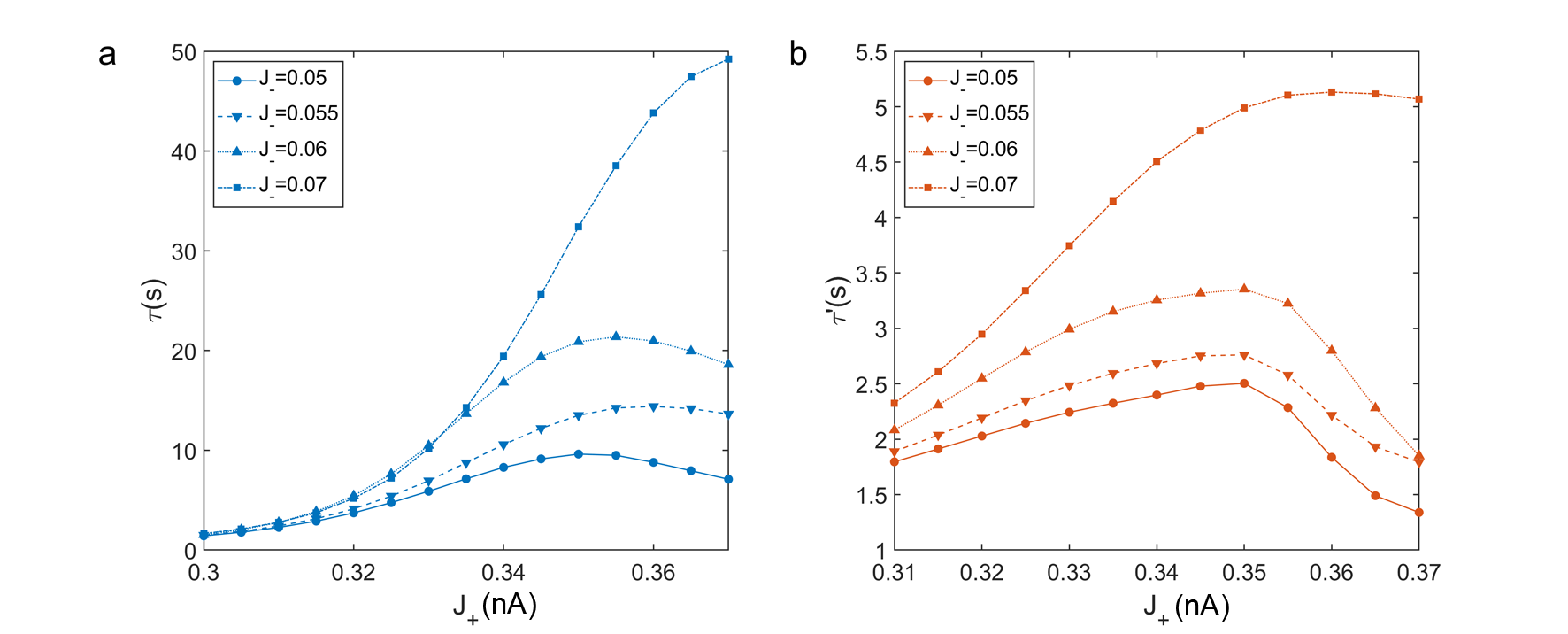

Supplement: S2 Fig — (a)The robustness against the random fluctuations and (b)The robustness against the distractors are greatly enhanced for further increasing mutual inhibition J−. (TIF) [file pcbi.1008209.s002.tif]
